# Case Report: Failure of eculizumab to block complement to prevent relapse of anti-phospholipid syndrome in kidney transplant recipient

**DOI:** 10.3389/fneph.2025.1572641

**Published:** 2025-06-18

**Authors:** Thibault Laban, Fredéric Pigneur, Constance Guillaud, Marie Agnès Dragon Durey, Houcine Hamidi, Caroline Pilon, Marc Michel, Nizar Joher, Philippe Grimbert, Hamza Sakhi, Antoine Morel, Marie Matignon

**Affiliations:** ^1^ Assistance Publique Hôpitaux de Paris (AP-HP), Groupe Hospitalo-Universitaire Chenevier Mondor, Nephrology and Renal Transplantation Department, Fédération Hospitalo-Universitaire “Innovative Therapy for Immune Disorders,”, Créteil, France; ^2^ Assistance Publique Hôpitaux de Paris (AP-HP), Groupe hospitalo-universitaire Chenevier Mondor, Radiology Department, Créteil, France; ^3^ Assistance Publique Hôpitaux de Paris (AP-HP), Groupe Hospitalo-Universitaire Chenevier Mondor, Internal Medicine Department, Fédération Hospitalo-Universitaire “Innovative Therapy for Immune Disorders,”, Créteil, France; ^4^ Université Paris Cité, INSERM UMRS 1138, Centre de Recherche des Cordeliers, Assistance Publique Hôpitaux de Paris (AP-HPP), Groupe Hospitalier Paris Centre, Hôpital Européen Georges Pompidou, Paris, France; ^5^ Assistance Publique Hôpitaux de Paris (AP-HP), Groupe Hospitalier Paris Centre, Hôpital Européen Georges Pompidou, Paris, France; ^6^ Univ Paris Est Creteil, INSERM IMRB U955, Créteil, France; ^7^ Assistance Publique Hôpitaux de Paris (AP-HP), Groupe Hospitalo-Universitaire Chenevier Mondor, Centre d’Investigation Clinique Biotherapy, Fédération Hospitalo-Universitaire “Innovative Therapy for Immune Disorders,”, Créteil, France

**Keywords:** kidney transplantation, antiphospholipid syndrome, eculizumab, thrombotic micro angiopathy, cortical necrosis

## Abstract

Catastrophic antiphospholipid syndrome (CAPS) leads to organ dysfunction due to thrombotic microangiopathy (TMA). Complement may play a role in CAPS, and its blockade could prevent antiphospholipid syndrome (APS) complications after kidney transplantation (KT). Here, we report a case of APS recurrence after KT in a 38-year-old woman with early acute cortical kidney allograft necrosis despite preventive eculizumab treatment, probably because of insufficient complement blockade. The patient had recurrent but controlled CAPS for years with renal dysfunction, leading to preemptive KT. Anticoagulation and eculizumab were administered to prevent thrombosis and TMA after KT. She developed acute kidney injury (AKI) with incomplete biological TMA. Imaging revealed cortical necrosis in the renal allograft. In the absence of donor-specific anti-HLA antibodies, we concluded a relapse. Additional doses of eculizumab and plasma exchange allowed the normalization of biological tests and improvement of kidney allograft function. A retrospective complement analysis showed an incomplete blockade at the time of AKI. One year after KT, the renal allograft function was impaired. This suggests that inadequate complement blockade leads to a relapse of APS in the renal allograft with cortical necrosis and dysfunction. Our case highlights the importance of monitoring complement activity and adjusting the dose of eculizumab or ravulizumab.

## Introduction

Catastrophic antiphospholipid syndrome (CAPS) is a thrombotic microangiopathy (TMA) that occurs in 1% of antiphospholipid syndrome (APS) (1). CAPS recurrence during surgery is common (40%) with a mortality rate of approximately 33% (1). Complement plays a key role in APS-mediated thrombosis (1). Eculizumab, an anti-C5 monoclonal antibody, has been used to prevent arterial thrombosis after kidney transplantation (KT) and refractory CAPS (2, 3).

Here, we report failure of eculizumab to prevent early acute cortical allograft necrosis after KT in a primary APS recipient.

A 38-year-old woman with primary APS underwent pre-emptive KT in April 2023. She started CAPS in 2005, which was associated with triple antiphospholipid (APL) positivity, renal TMA with cortical necrosis, and neurological and cardiac damage. She was treated with anti-vitamin K warfarin and relapsed in 2011, with suspected heparin-induced thrombocytopenia (HIT).

At KT, APL was undetectable and lupus anticoagulants were positive. C3 and C4 antigen levels were normal and soluble C5b-9 levels were low (205 ng/mL; reference value <300 ng/mL). Immunosuppressive therapy included anti-thymocyte globulin for past anti-HLA donor-specific antibodies (DSA), tacrolimus, mycophenolic acid, and steroids. Eculizumab was infused as follows: 1,200 mg before KT and 900 mg on day 1 and was planned to be infused weekly for four weeks (2). Surgery was performed with an INR of 2 and danaparoid sodium, as recommended (4). Danaparoid sodium anti-Xa target of 0.5 IU/mL to 0.7 IU/mL was achieved on day 1 and remained stable over the first 7 days. Serum creatinine level decreased to 145 µmol/L on day 5.

**Figure 1:**
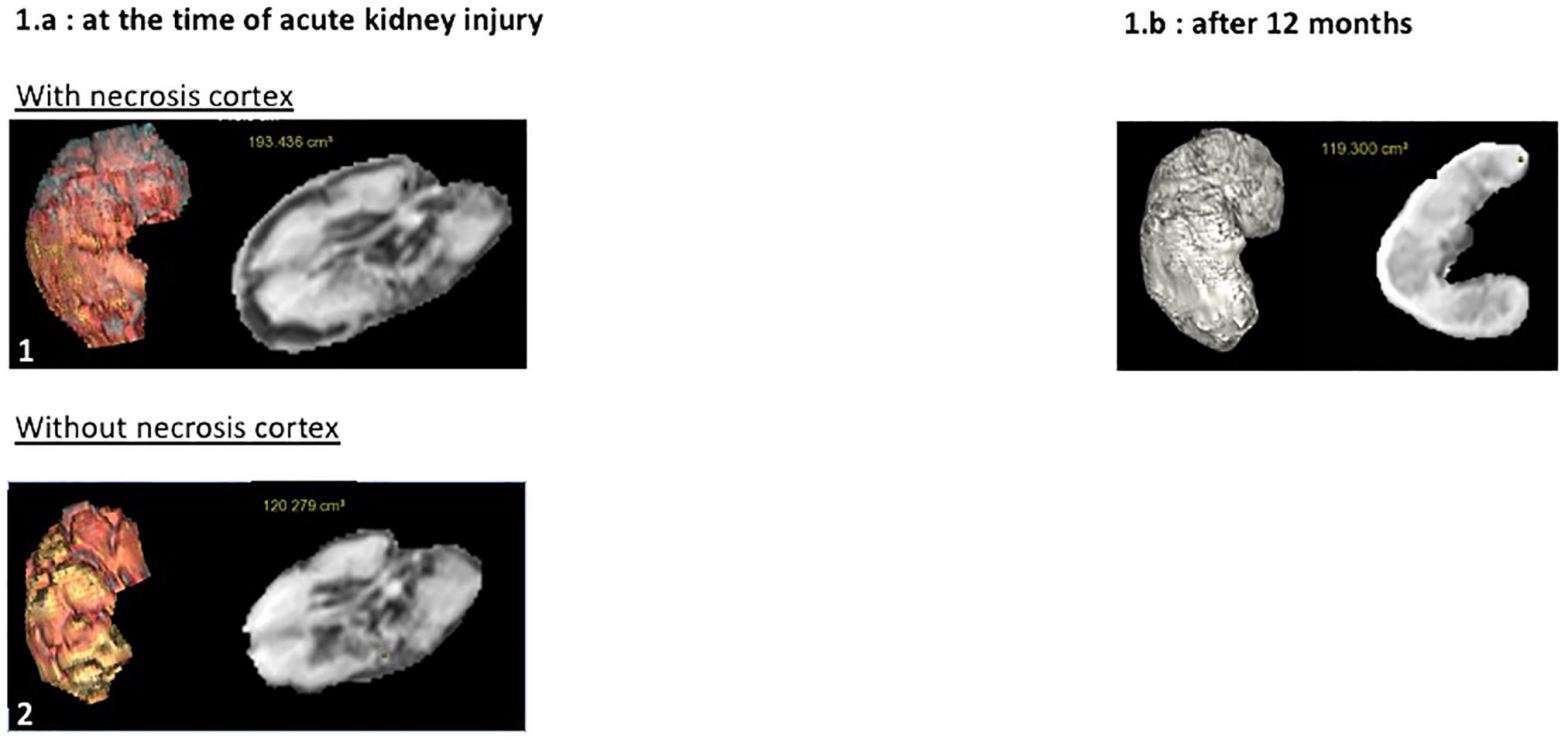
Contrast echography and magnetic resonance imaging (MRI). **(a)** Renal allograft contrast Doppler and contrast echography at the time of acute kidney injury. Renal allograft Doppler ultrasound showed no thrombosis of allograft vessels (1.a.1). Contrast echography revealed a cortical enhancement defect involving the entire allograft (1.a.2). **(b)** MRI at the time of acute kidney injury and at 12 months. At the time of acute kidney injury, MRI confirmed a cortical enhancement defect of the allograft measuring between 2.5 mm and 4.5 mm in thickness on the contrast-enhanced sequences, indicating cortical necrosis extending throughout the parenchyma, with approximately 50% of the cortex remaining vascularized, without venous or arterial thrombosis (1.b.1). At the 12-month follow-up, MRI showed atrophy of the cortical region, which exhibited a defect of enhancement on the contrast-enhanced sequences on the initial MRI, indicating sequelae of cortical necrosis resulting from the initial thrombotic microangiopathy (1.b.2).

On day 6, she had acute kidney injury, tubular proteinuria (proteinuria 317.6 mg/mmol creatininuria with 28.3 mg/mmol microalbuminuria), microscopic hematuria and a marked decrease in diuresis. The complement C3 and C4 fractions were normal, CH50 was undetectable, and soluble C5b-9 was slightly elevated (321 ng/mL; reference <300 ng/mL). Other results included anemia, thrombocytopenia, high LDH, normal haptoglobin, no schizocytes, negative APL serology, positive lupus anticoagulant, and tacrolimus residues between 4 ng/mL and 6 ng/mL. DSA was negative on day 6. Patients with vascular allograft thrombosis were excluded from the study (**Figure 1a**). Allograft contrast echography and magnetic resonance imaging (MRI) revealed cortical necrosis of the allograft (**Figures 1a, b**). We concluded that acute renal cortical necrosis was due to intragraft APS-induced TMA. We did not perform allograft biopsy because stopping anticoagulation for 48 h increases the risk of progression to CAPS.

**Figure 2:**
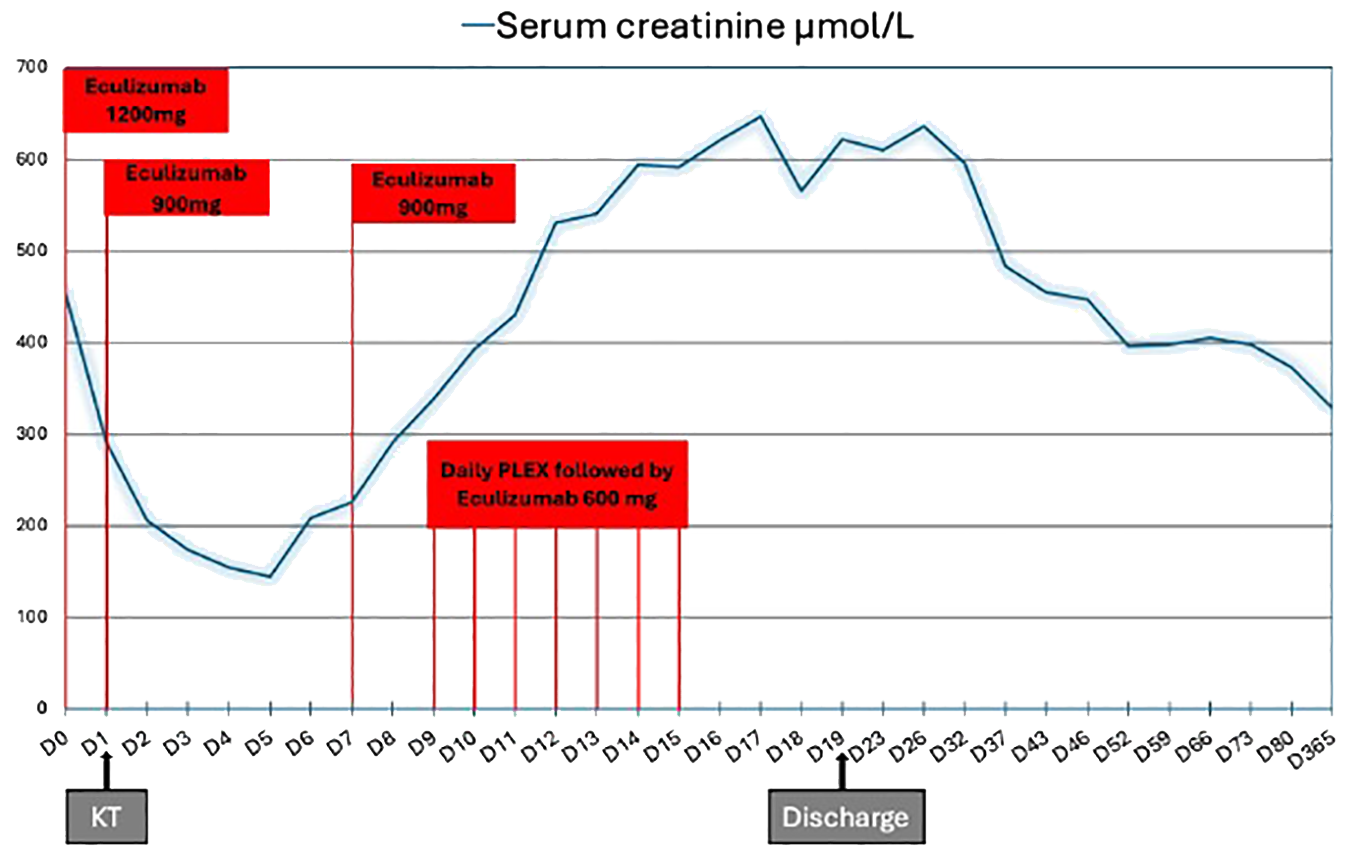
Kidney allograft function evolution and treatment. Eculizumab was infused as follows: 1,200 mg before kidney transplantation (KT) and 900 mg on day 1. Serum creatinine decreased to 145 µmol/L on day 5. On day 6, she received an extra 900 mg dose of eculizumab, high-dose steroids, plasma exchange (PLEX) and eculizumab 600 mg within 60 min after the end of each PLEX session, switching from tacrolimus to belatacept and warfarin. On day 17 peak creatinine was 647 µmol/L. Eculizumab was stopped three weeks after KT without relapse. KT, kidney transplantation; PLEX, plasma exchange.

**Figure 3:**
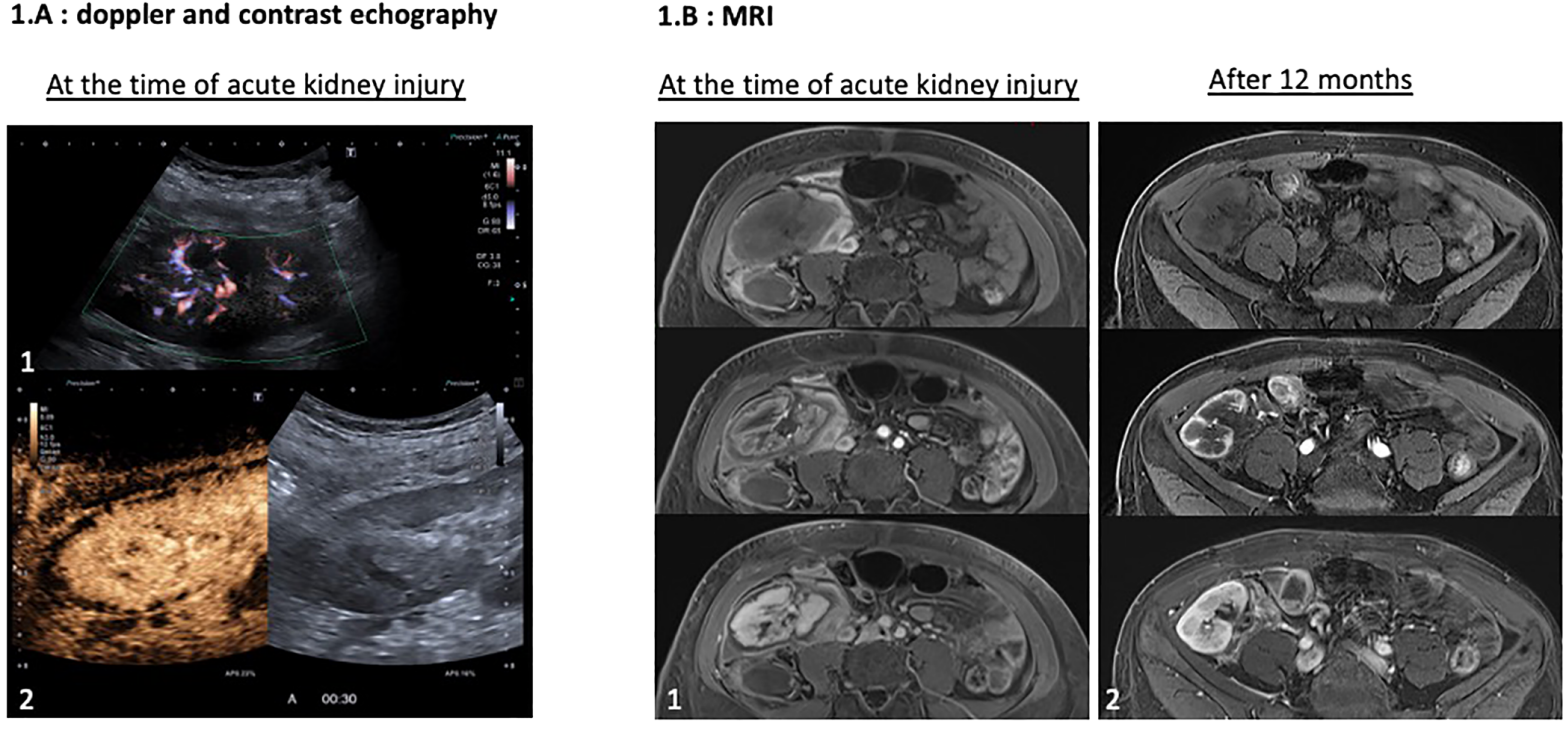
Evolution of renal allograft volumetry; **(A)** at the time of diagnosis of cortical necrosis and **(B)** after 12 months. The volume of the allograft appears to decrease significantly from the time of acute kidney injury (2.a.1: 193 cm³) to 12 months later (2.b: 119 cm³), representing a 38% reduction in renal volume, mainly affecting the renal cortex. In fact, the volume of the kidney allograft remained similar between 120 (2.a.1) and 119 cm³ (2.b), at both time points when cortical necrosis was not taken in account for volumetry during the acute kidney injury, demonstrating that it was the necrotic part that underwent atrophy.

Treatment included an additional 900 mg dose of eculizumab, high-dose steroids, plasma exchange (PLEX), switching from tacrolimus to belatacept, and warfarin (4). On day 17 peak creatinine level was 647 µmol/L. Anemia and thrombocytopenia normalized, and the creatinine level decreased to 400 µmol/L. CH50 remained blocked during PLEX and serum C5b-9 levels were less than 300 ng/mL. Eculizumab was stopped three weeks after KT without relapse. **Figure 2** summarizes treatment and biological evolution. One year after transplantation, creatinine was 328 µmol/L, proteinuria 2.15 g/g, microalbuminuria 1.59 g/g and DSA negative. APL serology was negative, and the lupus anticoagulant test results were positive. Renal scintigraphy revealed residual fixation of both native kidneys and good allograft fixation with vascular invasion. MRI of the allograft revealed significant cortical atrophy of the cortical zone, which was necrotic on the initial MRI (**Figure 3**).

We retrospectively analyzed complement activation products and components (**Table 1**) (5). The complement was activated before KT and was relatively blocked after KT. C5a levels decreased after treatment. C3a and sC5b9 levels slightly increased, while the residual plasma concentration of eculizumab was too low. This suggests that the eculizumab dose was insufficient to ensure complete complement blockade.

**Table 1:** Analysis of complement activation products (C3a, C5a, s-C5b9, Ba, and Bb) and components (Factor H and Factor I).

	Before transplantation	At the time of the relapse	Normal values, Median [IQR]
Ba (ng/ml)	>3,206.00	>3,206.00	451.2 [394.7, 550.7]
Bb (ug/ml)	2.29	1.68	1.2 [0.9, 1.5]
C3a (ng/ml)	191.66	231.69	53.0 [37.7, 71.3]
C5a (ng/ml)	57.24	13.61	6.1 [4.8, 8.9]
sC5b9 (ng/mL)	205	321	<300
Factor H (ug/ml)	320.51	286.54	351.1 [298.6, 409.7]
Factor I (µg/ml)	23.53	31.18	20.97 [16.66, 26.14]

Retrospective whole exome analysis found no complement gene mutations.

Ba levels are highly dependent on renal function.

## Discussion

Here, we report failure of eculizumab to prevent acute renal allograft cortical necrosis after KT in a recipient with primary APS and a history of CAPS. The clinical context led us to conclude that acute renal allograft TMA was due to APS relapse. With APS treatment and enhanced complement blockade, TMA stigmata decreased, and renal allograft function improved.

We considered APS-induced intragraft TMA. KT induces endothelial injury due to ischemia–reperfusion, alloantibodies, and autoantibodies (6), and endothelial dysfunction is a key pathological component of APS relapse (1). Second, switching from anticoagulant therapy to heparin is risky in CAPS (1). Finally, we did not consider ABMR to be the cause of TMA (7). In the era of modern anti-HLA detection using Luminex assays, a case of complete cortical necrosis induced by early ABMR without DSA at the time of transplantation has been reported to date in the presence of non-HLA antibodies (ATR1 antibodies) after preemptive ABO-incompatible kidney transplantation (8). The most common clinicopathological presentation of ATR1 induced ABMR include TMA but no cortical necrosis (9–11). Endothelial cell crossmatch can exclude non-HLA antibody-induced cortical necrosis; however, this test is not routinely available (9). The absence of DSA in ABMR has been reported to be associated with better prognosis (12). Thus, APS-induced TMA appears more likely than ABMR, although we could not definitively exclude this diagnosis.

**Figure 4:**
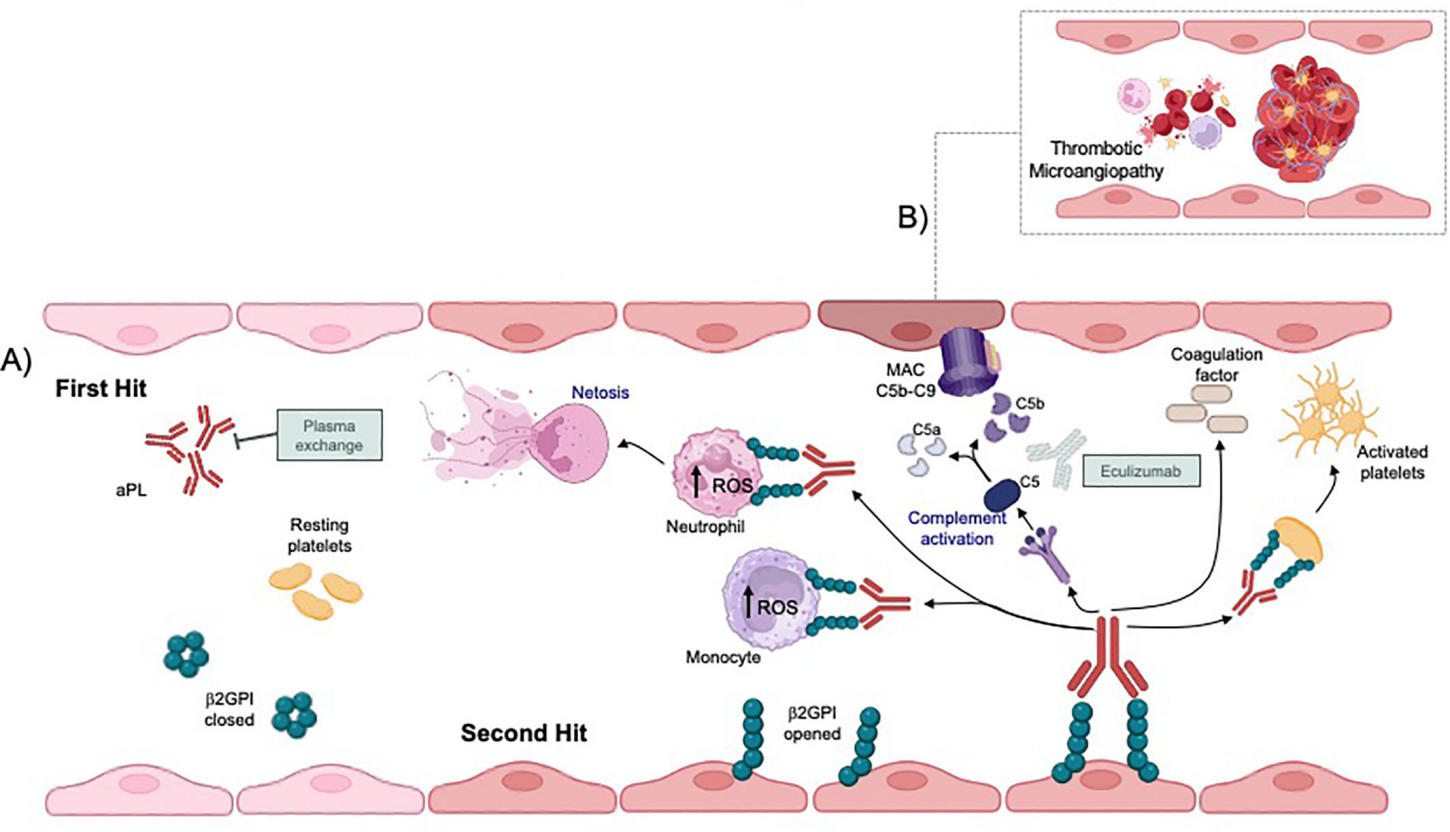
Potential pathogenesis of antiphospholipid antibodies leading to thrombotic microangiopathy and possible complement activation after kidney transplantation. **(A)** The pathophysiology of antiphospholipid syndrome (APS) could be explained by the “two-hit hypothesis,” in which aPL acts as the initial catalyst in creating a prothrombotic state. The plasma exchange (PLEX) could significantly reduce the concentration of aPL thereby reducing the prothrombotic state. Kidney transplantation could be the second strike causing endothelial cell damage that disrupts the natural anticoagulant system. aPL-mediated endothelial cell dysfunction activates endothelial cells, platelets, monocytes, and neutrophil promoting neutrophil extracellular trap release and tissue factor expression as well as complement cascade activation. **(B)** The complement-mediated tissue factor plays a critical role in the pathogenesis of thrombotic microangiopathy in APS; therefor treatment with complement inhibitors in addition to anticoagulation may reduce renal injury. MAC, Membranous Attack Complex.

Eculizumab partially failed to prevent KT-induced APS relapse. Blocking C5 may prevent APS- and CAPS-associated posttransplant complications (2). Renal thrombosis is a major complication of KT in patients with a history of APS and/or CAPS. It can be prevented with anticoagulation and eculizumab (2, 13). aPLs mice deficient in C3, C5, or C6, or treated with complement inhibitors, have fewer episodes of thrombosis (14). Successful complement blockade has been reported in three resistant APS-induced TMA relapses after KT, but intragraft C5b-9 deposits persist (15). Complement appeared to be over-activated in CAPS, and APLs induced and increased the deposition of C5b-9 *in vitro* (a biological indicator of complement activation) (16, 17). Outside KT, one report described refractory CAPS that was resistant to complement blockade (3). Despite the small sample size in this report, hematological disturbances improved more frequently, and early treatment with eculizumab seemed to prevent renal cortical necrosis. In our patient, APS relapsed without complete CAPS, and we hypothesized that complement blockade was insufficient to prevent renal cortical necrosis but that eculizumab may have limited APS reactivation to the allograft. **Figure 4** presents the presumed physiopathology of complement activation in APS relapse and the mechanisms of action of eculizumab and PLEX.

Complement analysis showed complement blockade at relapse, highly soluble C5b-9, and retrospectively high C3a levels. Higher sC5b-9 levels have been associated with relapse of hemolytic and uremic syndromes after eculizumab discontinuation (18). Taken together, these results suggest that the eculizumab dose was too low. To avoid relapse, we suggest early and close complement monitoring after KT and dose adjustment to sC5b-9 and/or other complement fractions or instead of eculizumab, ravulizumab, a new complement C5 inhibitor that provides more immediate, complete, and sustained C5 inhibition (19). The use of inhibitors involved in the complement cascade should also be considered.

Studies on rare germline variants in complement genes have not revealed any mutations in this patient. However, they may be of interest in CAPS as these patients are likely to have a higher rate of complement activation (16). The question of differential responses to eculizumab according to the rate of mutations requires further investigation.

In conclusion, we report here the failure of complement blockade to prevent complete recurrence of APS after KT in patients with cortical necrosis and chronic kidney disease. In patients with CAPS, anticoagulation and complement blockade prevent renal thrombosis and TMA post-transplantation. Our hypothesis is that inadequate complement blockade should be monitored before and after KT, with subsequent adjustment of the eculizumab dose or the use of ravulizumab.

## Data Availability

The raw data supporting the conclusions of this article will be made available by the authors, without undue reservation.
